# Impairment of Mitochondria in Adult Mouse Brain Overexpressing Predominantly Full-Length, N-Terminally Acetylated Human α-Synuclein

**DOI:** 10.1371/journal.pone.0063557

**Published:** 2013-05-07

**Authors:** Theodore A. Sarafian, Christopher M. Ryan, Puneet Souda, Eliezer Masliah, Upendra K. Kar, Harry V. Vinters, Gary W. Mathern, Kym F. Faull, Julian P. Whitelegge, Joseph B. Watson

**Affiliations:** 1 Department of Psychiatry & Biobehavioral Sciences, David Geffen School of Medicine at UCLA, Los Angeles, California, United States of America; 2 Department of Neuroscience, University of California, San Diego School of Medicine, La Jolla, California, United States of America; 3 Department of Pathology and Laboratory Medicine, David Geffen School of Medicine at UCLA, Los Angeles, California, United States of America; 4 Department of Neurosurgery, David Geffen School of Medicine at UCLA, Los Angeles, California, United States of America; Ecole Polytechnique Federale de Lausanne (EPFL), Switzerland

## Abstract

While most forms of Parkinson’s Disease (PD) are sporadic in nature, a small percentage of PD have genetic causes as first described for dominant, single base pair changes as well as duplication and triplication in the α-synuclein gene. The α-synuclein gene encodes a 140 amino acid residue protein that interacts with a variety of organelles including synaptic vesicles, lysosomes, endoplasmic reticulum/Golgi vesicles and, reported more recently, mitochondria. Here we examined the structural and functional interactions of human α-synuclein with brain mitochondria obtained from an early, pre-manifest mouse model for PD over-expressing human α-synuclein (ASOTg). The membrane potential in ASOTg brain mitochondria was decreased relative to wildtype (WT) mitochondria, while reactive oxygen species (ROS) were elevated in ASOTg brain mitochondria. No selective interaction of human α-synuclein with mitochondrial electron transport complexes cI-cV was detected. Monomeric human α-synuclein plus carboxyl terminally truncated forms were the predominant isoforms detected in ASOTg brain mitochondria by 2-dimensional PAGE (Native/SDS) and immunoblotting. Oligomers or fibrils were not detected with amyloid conformational antibodies. Mass spectrometry of human α-synuclein in both ASOTg brain mitochondria and homogenates from surgically resected human cortex demonstrated that the protein was full-length and postranslationally modified by N-terminal acetylation. Overall the study showed that accumulation of full-length, N-terminally acetylated human α-synuclein was sufficient to disrupt brain mitochondrial function in adult mice.

## Introduction

The causes and cures for Parkinson’s Disease (PD) remain elusive, but many roads of investigation have led to the critical importance of the α-synuclein protein. The α-synuclein gene encodes a 140 amino acid residue protein that is expressed ubiquitously in the brain and is enriched in presynaptic terminals [1], [2]. The α-synuclein protein exists mostly as an unfolded soluble monomer of ∼14 kDa, but it can assume an amphipathic, α-helical conformation when bound to acidic phospholipids in a variety of organelles including lysosomes, mitochondria, and endoplasmic reticulum/Golgi vesicles [3]–[8]. In keeping with a presynaptic function, both mutant and over-expressed forms of normal human α-synuclein interact with synaptic vesicles at presynaptic terminals and have been shown to negatively impact synaptic vesicle function, likely at a step prior to docking and upstream of the pool of vesicles poised for rapid neurotransmitter release [9]–[13].

The A53T amino acid substitution in the full-length 140 amino acid sequence of human α-synuclein was the first PD familial mutation identified, while at least two additional inherited forms with single amino acid mutations (A30P, E46K) have been identified subsequently [14]. More recently genome-wide association studies linked genetic variants for the α-synuclein (*SNCA*) locus to the more widespread forms of idiopathic PD. Moreover the identification of α-synuclein gene duplication and triplication in additional families with inherited PD suggest that not only mutant forms of α-synuclein but also over-expressed forms are major contributing factors in PD. Numerous types of posttranslational modifications (PTMs) of the mature α-synuclein protein have also been identified in sporadic forms of PD (e.g. acetylation, phosphorylation, nitration, oxidation, ubiquitination, SUMOylation, truncation) [15]. The α-synuclein protein ultimately accumulates as insoluble fibrils in Lewy bodies in sporadic forms of PD [16] and in most inherited forms of PD including the most common forms with leucine-rich repeat kinase 2 (*LRRK2*) gene mutations [17].

More recent reports show that human α-synuclein is localized in mitochondria of the substantia nigra and striatum from PD postmortem tissue where it can disrupt complex I (cI) of the electron transport chain [18]. These results are consistent with previous reports that mitochondrial cI activity is reduced in PD [19] and cI is a prime target of environmental toxins such as 1-methyl-4-phenyl-1, 2, 3, 6-tetra-hydropyridine (MPTP) and rotenone that inhibit respiratory energetics and produce oxidative stress [20]–[22]. cI activity is also disrupted in PD rodent models expressing either wildtype (WT) or mutant forms of human α-synuclein [23]–[26]. Other mitochondrial changes include association with the adenylate translocator as well as mitochondrial enlargement, vacuolation, fragmentation, and degeneration [27]–[30].

Here we examined the structural and functional interactions of human α-synuclein with brain mitochondria obtained from an early, pre-manifest mouse model for PD over-expressing human α-synuclein (ASOTg) [31]–[35]. Overall it appears that overexpression of an N-terminally acetylated, monomeric form plus minor truncated forms of human α-synuclein are sufficient to impair brain mitochondrial function in adult mice.

## Results

### Overexpressed Human α-Synuclein is Closely Associated with Brain Mitochondria

**Figure 1:**
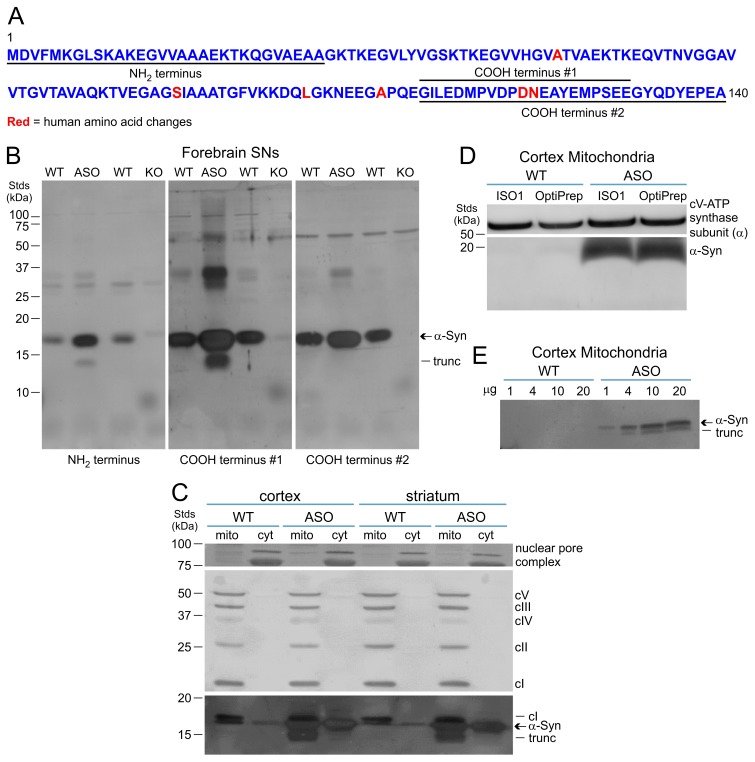
Isolation of overexpressed human α-synuclein and truncated forms with brain mitochondria. **A.** The entire amino acid sequence of human α-synuclein (140 amino acid residues) is displayed. Epitope stretches of amino acids used to raise human α-synuclein-selective antibodies are underlined (NH_2_ terminus, COOH terminus #1, COOH terminus #2). Human substitutions (total of 6) in the α-synuclein amino acid sequence are indicated in red. **B.** Western immunoblotting detected both endogenous mouse α-synuclein and overexpressed human α-synuclein **(αSyn)** (15–20 kDa) in synaptoneurosome (SN) fractions (20 µg) from WT and ASOTg littermates respectively using human α-synuclein-selective antibodies (left panel, NH_2_ terminus; middle panel, COOH terminus #1; right panel, COOH terminus #2). WT and *Snca^−/−^*littermates (KO) lacking mouse α-synuclein served as additional controls. Smaller forms (<15 kDa) of human α-synuclein were also detected in ASOTg SNs (left/middle panels) but not with the COOH terminus #2 antibody (right panel), consistent with carboxyl terminal truncation. **C.** Segments of the same immunoblot with brain (cortex, striatum) mitochondria (mito) and cyotosolic (cyt) fractions (20 µg each) from both WT and ASOTg mice were successively probed with different antibodies based on the size of the target protein. Target proteins included: nuclear pore complex proteins (top panel), subunit proteins for each of the five mitochondrial electron transport complexes (cI–cV) complexes (middle panel), and human α-synuclein (ASOTg) (bottom panel). Since blot segments were not stripped of immunoreactivity between antibody reprobing, electron transport complex cI band was detected as a residual band above α-synuclein. **D.** Human α-synuclein was also detected in purified cortex mitochondria (20 µg) isolated from ASO transgenic mice by the ISO1 protocol and a subsequent step using ultracentrifugation and density gradients (OptiPrep). cV-ATP synthase subunit (α) served as control for mitochiondria enrichment. **E.** Truncated forms of human α-synuclein were detected with increasing concentrations (4–20 µg) of ASOTg mitochondria but not with WT mitochondria.

Because α-synuclein is predominantly a presynaptic protein [1], [2], we first used forebrain synaptoneurosomes (SNs) [36] to characterize domain-selective antibodies for human α-synuclein (NH_2_ terminus, COOH terminus #1, COOH terminus #2) (Fig. 1A). Each antibody detected elevated levels of human α-synuclein (15–20 kDa) in ASOTg relative to WT SNs; each antibody also has some cross-immunoreactivity with endogenous WT mouse α-synuclein (Fig. 1B). No selective immunoreactivity was detected in SN fractions from α-synuclein KO mice used as negative controls. Smaller α-synuclein forms were also detected in ASOTg SNs (left/middle panels). Overall the immunoblotting patterns were consistent with carboxyl terminal truncation, since the COOH terminus #2 antibody (only epitope containing the last 9 amino acids at carboxyl terminus) failed to detect smaller forms (right panel). Truncated forms were not detected in WT SNs, but this may be due to their lower amounts. Larger molecular weight forms (>20 kDa) in the ASOTg lanes appeared to be unrelated cross-immunoreactive bands based on side-by-side comparisons to WT and KO lanes.

In light of a previous report showing that human α-synuclein was localized with ASOTg midbrain mitochondria [30], we also asked if human α-synuclein was enriched in forebrain (striatum, cortex) mitochondrial fractions from ASOTg mice (Fig. 1C). For these experiments and all subsequent experiments, we used the α-synuclein COOH terminus #1 antibody that can detect both monomeric and carboxyl terminally truncated forms. As expected, nuclear pore complex proteins were lacking in brain mitochondrial fractions, but a residual amount was found in cytosolic fractions (top panel). Importantly enriched amounts of the appropriate subunit protein in each of the five mitochondrial electron transport complexes (cI–cV) complexes were detected in both cortex and striatum mitochondria fractions relative to cytosolic fractions from both WT and ASOTg mice (middle panel). Both human α-synuclein (ASOTg) and endogenous mouse α-synuclein (WT) were enriched in mitochondria relative to cytosolic fractions (bottom panel). However, truncated forms of α-synuclein were mainly detected in ASOTg mitochondrial fractions, but not discernible in WT fractions (bottom panel).

Using an additional purification step for cortex mitochondrial isolation by density gradient purification (MITOSIO1 plus OptiPrep, Fig. 1D), we again detected elevated levels of mitochondrial human α-synuclein in ASOTg relative to WT fractions along with cV-ATP synthase subunit α as a mitochondrial marker. Here truncated forms of human α-synuclein were not detected in cortex mitochondria suggesting that their appearance was concentration-dependent. This idea was borne out when truncated human α-synuclein was detected with increasing intensity in larger amounts of ASOTg cortex mitochondrial fractions (see Fig. 1E). Overall some brain mitochondrial human α-synuclein was carboxyl terminally truncated, consistent with similar observations in whole brain studies [37]–[39].

### Impairment of Brain Mitochondria from ASOTg Mice Overexpressing Human α-Synuclein

#### Mitochondrial Membrane Potential

**Figure 2:**
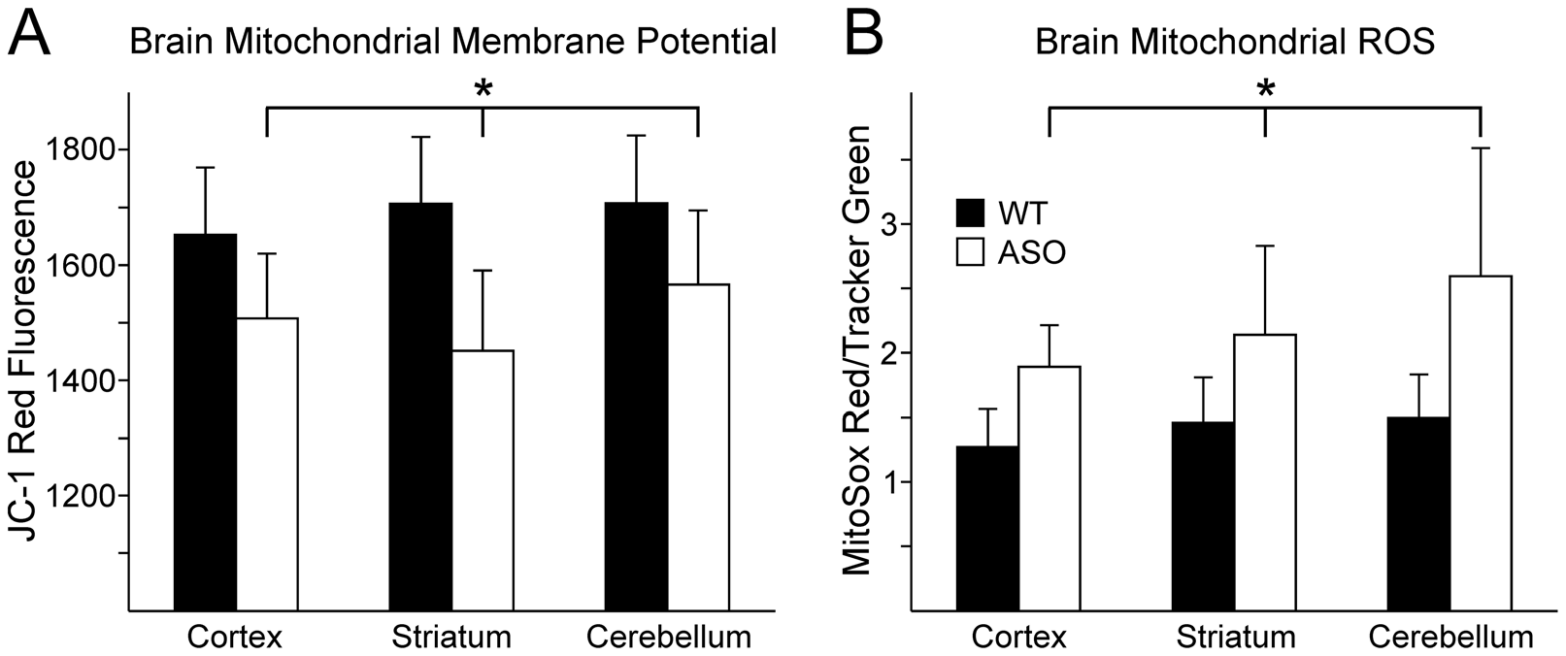
Overexpressed human α-synuclein disrupted brain mitochondrial functions. **A.** The mitochondrial membrane potential, detected as JC-1 red fluorescence, was reduced in all ASOTg brain region mitochondria (cortex, striatum, cerebellum), and was significantly different from WT values when all brain regions were considered (Two Way ANOVA, P = 0.013)(* indicates that all brain regions must be included for significance between genotype, WT vs ASOTg). All values are expressed as mean ± Standard Error of Mean (SEM). However, Bonferroni post-hoc analyses showed no difference in membrane potential between brain regions [F (2,52) = 0.205, P = 0.815]. FCCP (10 µg/ml) decreased greater than 95% of membrane potential on average in separate experiments to confirm authentic mitochondrial membrane potential. **B.** Mitochondrial reactive oxygen species (ROS) were significantly elevated in all ASOTg brain regions relative to WT brain regions (Two Way ANOVA, P = 0.044) (* indicates that all brain regions must be included for significance between genotype, WT vs ASOTg). There was no difference in ROS between brain regions [F (2,30) = 0.156, P = 0.857].

Since it has been widely reported that human α-synuclein disrupts brain mitochondrial function [40], we examined the membrane potential in WT and ASOTg brain mitochondria (Fig. 2). The membrane potential (JC-1 red fluorescence alone; assumed little or no cytosol) was significantly reduced in all ASOTg mitochondria relative to WT mitochondria (Two Way ANOVA, P = 0.031) (Fig. 2A). However, Bonferroni post-hoc *t*-tests showed no difference in membrane potential between brain regions (WT, mean ± Standard Error of Mean (SEM): cortex, 1652.8±90.0, N = 11; striatum, 1706.1±88.5, N = 9; cerebellum, 1706.9±101.4, N = 9)(ASOTg: cortex, 1507.7±67.8, N = 11; striatum, 1451.1±78.5, N = 9; cerebellum, 1566.7±158.0, N = 9 [F(2,52) = 0.205, P = 0.815]. Taken together the data show that mitochondria throughout the brain of adult mice have impaired membrane potential when exposed to overexpressed human α-synuclein.

#### Mitochondrial ROS

PD has been closely associated with oxidative stress measured as elevated ROS levels [20], [21], [40]. To determine if overexpressed human α-synuclein increases brain oxidative stress, we measured ROS levels in WT and ASOTg brain mitochondria using a mitochondria-specific probe MitoSox Red.

Mitochondrial ROS levels were significantly increased in all ASOTg brain regions’ mitochondria relative to WT (Two Way ANOVA, P = 0.044) (Fig. 2B). However, there was no difference in ROS between brain regions (WT, mean ± SEM: cortex, 1.27±0.24; striatum, 1.46±0.29; cerebellum, 1.50±0.28, N = 6 for all regions) (ASOTg: cortex, 1.89±0.29; striatum, 2.14±0.57; cerebellum, 2.60±0.82, N = 6 for all regions) [F(2,30) = 0.156, P = 0.857]. Overall ASOTg brain mitochondria exhibit significant oxidative stress in the face of increased human α-synuclein.

### No Selective Association of Elevated Human α-Synuclein with Mitochondrial Electron Transport Complexes

**Figure 3:**
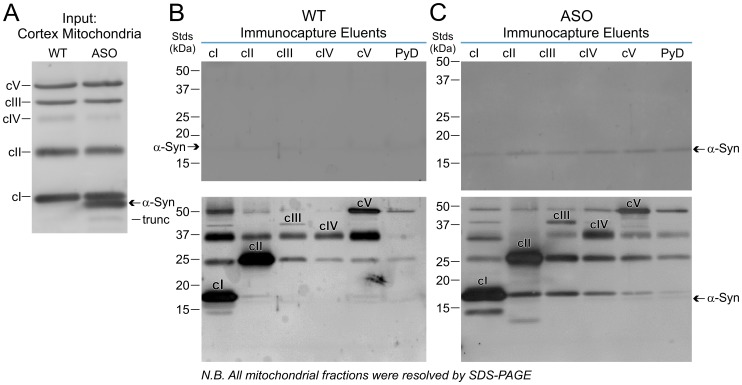
Non-specific association of human α-synuclein with cortex mitochondrial complexes by immunocapture. **A.** SDS-PAGE and Western immunoblotting with a total OXPHOS antibody cocktail detected the appropriate subunit protein equally in each of the five mitochondrial electron transport complexes (cI–cV) complexes from WT and ASOTg mitochondrial fractions. Re-probed blot confirmed elevated human α-synuclein in the ASOTg mitochondrial fraction; minor lower band may correspond to COOH-terminal truncated or alternative spliced forms of α-synuclein. **B.** Negligible levels of WT mouse α-synuclein were recovered as eluents from antibody-conjugated beads selective for electron transport complexes cI–cV and pyruvate dehydrogenase (PyD) after extensive washing with lauryl maltoside detergent-containing buffer. Reprobing of blots with the total OXPHOS antibodies confirmed relative enrichment for immunocapture of each representative protein (see labeled bands in bottom panel). Labeling in the PyD fraction represents background immunocapture. **C.** Higher levels of human α-synuclein were recovered from ASOTg fractions relative to WT (top panel) but were represented equally in all immunocaptured complexes cI–cV, consistent with non-selective association (experiment was repeated 2 times). Human α-synuclein was also observed in PyD, a soluble complex not associated with inner mitochondrial membrane, in amounts roughly similar to those from the cI–cV complexes. Reprobing of blots with the total OXPHOS antibodies confirmed relative enrichment for immunocapture of each representative protein.

Lower membrane potentials and higher ROS in ASOTg brain mitochondria could emanate from a variety of membrane targets compromised by human α-synuclein. One likely candidate is the mitochondrial electron transport complex c1 [18], [19]. Here we examined the association of α-synuclein with cI as well as other electron transport complexes cII-cV in brain mitochondria by immunocapture experiments. Staining with a total OXPHOS antibody cocktail for unique protein subunits in each of the five complexes revealed that the appropriate complex-selective protein was represented equally in WT and ASOTg cortex mitochondrial fractions resolved by SDS-PAGE (Fig. 3A). As expected, the re-probed blot detected a relatively higher amount of human α-synuclein in the ASOTg mitochondrial fraction, while a minor amount of truncated α-synuclein was also evident.

For WT immunocapture experiments, only negligible levels of α-synuclein were recovered from each of the five complexes after extensive washing with lauryl maltoside detergent-containing buffer (Fig. 3B). Although human α-synuclein was recovered at higher levels in comparable ASOTg fractions, it was represented equally in all immunocaptured complexes, consistent with non-selective association (Fig. 3C, top panel)(repeated twice for ASOTg mitochondria). Truncated forms were not apparent. Reprobing of blots with the total OXPHOS antibodies confirmed relative enrichment for each representative complex protein (Fig. 3B/C, bottom panel). There was some cross-immunoreactivity amongst the complexes, which may reflect isolation of integral membrane “supercomplexes” that can occur under certain conditions [41].

We also examined the association of α-synuclein with the pyruvate dehydrogenase (PyD) complex, which is a soluble complex found in the mitochondrial matrix and not associated with the inner membrane. A similar amount of human α-synuclein was also observed in PyD immunocaptured samples (Fig. 3C, last lane), suggesting that the presence of α-synuclein did not result from interaction with residual lipid associated with membrane-bound complexes. Rather this may reflect some degree of non-selective retention by the immunocapture beads.

### Human α-Synuclein Associates with Brain Mitochondria Mainly as a Monomer with Minor Truncated Species

**Figure 4:**
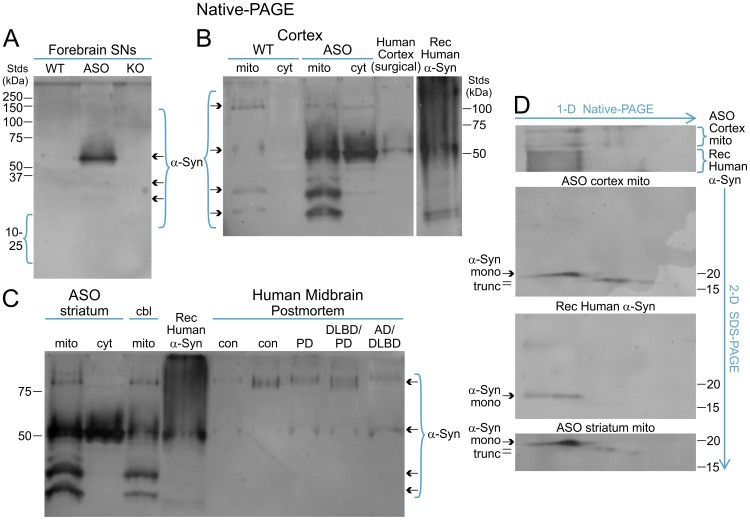
Brain synaptic and mitochondrial human α-synuclein were resolved mainly as a monomeric protein with truncated species by Native-PAGE and 2-dimensional PAGE (Native/SDS) immunoblotting. A. An α-synuclein (αSyn)-immunoreactive band of 50–60 kDa plus smaller forms (arrows, bracketed) were detected in sonicated ASOTg (ASO) forebrain SNs (10 µg) by Native-PAGE/immunoblotting. An SN fraction from *Snca^−/−^*mice (KO) served as a negative control. B. As a positive control for monomer conformations, sonicated recombinant human α-synuclein (Rec Human αSyn)(0.2–0.4 µg) was run on a Native gel and detected by immunoblotting (last lane). Recombinant α-synuclein was resolved as a diffuse band of 50–60 kDa amongst a large smear of bands > than 50 kDa half way up the gel (bracketed). WT and ASOTg cortex mitochondria (mito) and cytosolic (cyt) fractions (20 µg) as well as surgically resected human cortex (20 µg) were resolved on the same blot side by side with recombinant α-synuclein. A major band of 50–60 kDa is resolved but smaller bands were also detected mainly in the ASOTg mitochondrial fraction. C. In a separate Native-PAGE/blotting experiment, a major band of 50–60 kDa was also detected in ASOTg striatum mitochondrial/cytosolic fractions, a cerebellum mitochondrial preparation, recombinant α-synuclein, and five human postmortem midbrain tissues [con (control Case #1), con (control Case #2), PD (Case #4), DLBD/PD (Case #3), AD/DLBD (Case #5). D. Cortex mitochondrial and recombinant human α-synuclein were resolved by two-dimensional PAGE (1-D Native/2-D SDS) and immunoblotting. Native-PAGE isoforms (≥50 kDa) of recombinant α-synuclein were resolved mainly as monomers (mono) close to 20 kDa in size on SDS-PAGE. Similar sized Native-PAGE isoforms (≥50 kDa) observed in ASOTg cortex mitochondrial fractions were also resolved as monomers on SDS-PAGE. Smaller mitochondrial forms (trunc) closer to 15 kDa in size were also detected supporting their COOH-terminal truncation. In a separate 2-D experiment, α-synuclein monomers and truncated forms were also observed in an ASOTg striatum mitochondrial fraction (only 2-D SDS-PAGE shown)(bottom panel).

Overall the immunocapture data show that α-synuclein does *not* selectively associate with either inner membrane or soluble complexes localized within ASOTg mitochondria, at least at this adult age (2–4 months). However the use of detergents in the immunocapture and SDS-PAGE/immunoblotting experiments preclude the identification of higher order oligomeric/fibril forms of α-synuclein due to potential detergent-induced structural alterations [42]–[45]. To circumvent this problem, we sonicated WT and ASOTg mitochondria to lyse membranes and resolved proteins by Native-PAGE/immunoblotting (Fig. 4). We first examined SN fractions, where α-synuclein was detected as an immunoreactive band of 50–60 kDa plus minor amounts of smaller forms (arrows) in ASOTg forebrain SNs, but not in WT or KO control samples. As a positive control for unfolded monomer conformations, recombinant human α-synuclein was also examined in side-by-side comparison with WT and ASOTg cortex mitochondria and cytosolic fractions as well as a homogenate prepared from surgically resected human cortex (Fig. 4B). Recombinant α-synuclein was resolved as a diffuse band of 50–60 kDa with some smaller bands and a smear of larger bands half way up the blot. A similar sized major band of 50–60 kDa and a smear of larger forms were also detected in the WT/ASOTg brain fractions and in resected human cortex. However, smaller bands were only detected in the mouse brain mitochondrial fractions (both WT and ASOTg). In a separate experiment (Fig. 4C), both ASOTg striatum and cerebellum mitochondria also revealed a 50–60 kDa α-synuclein band plus smaller forms; both control and PD-related human postmortem midbrain tissues contained only the 50–60 kDa form and some larger forms.

The Native-PAGE experiments suggested that both endogenous mouse and overexpressed human α-synuclein were resolved as 50–60 kDa tetrameric isoforms, while the smaller forms found selectively in mitochondrial fractions were monomer-dimer-trimer combinations. To test this proposal further, cortex mitochondria and recombinant human α-synuclein were resolved by two-dimensional PAGE (Native/SDS) and immunoblotting (Fig. 4D). As expected, the 50–60 kDa isoform as well as larger forms previously observed on Native-PAGE for both ASOTg brain mitochondria and recombinant α-synuclein were resolved mainly as monomers between 15–20 kDa in size on SDS-PAGE (middle panels). Surprisingly the smaller forms were detected not as monomers but rather as lower molecular weight forms, thereby ruling out their oligomeric nature and supporting their truncation. In a separate experiment, this result was reproduced using a ASOTg striatum mitochondria fraction (bottom panel, Fig. 4D). Overall we concluded that overexpressed human α-synuclein as well as endogenous mouse α-synuclein was associated with brain mitochondria mainly as soluble monomeric forms with some degree of carboxyl terminal truncation.

### No Immunoreactivity of Mitochondrial Human α-Synuclein with Amyloid Conformational Antibodies

**Figure 5:**
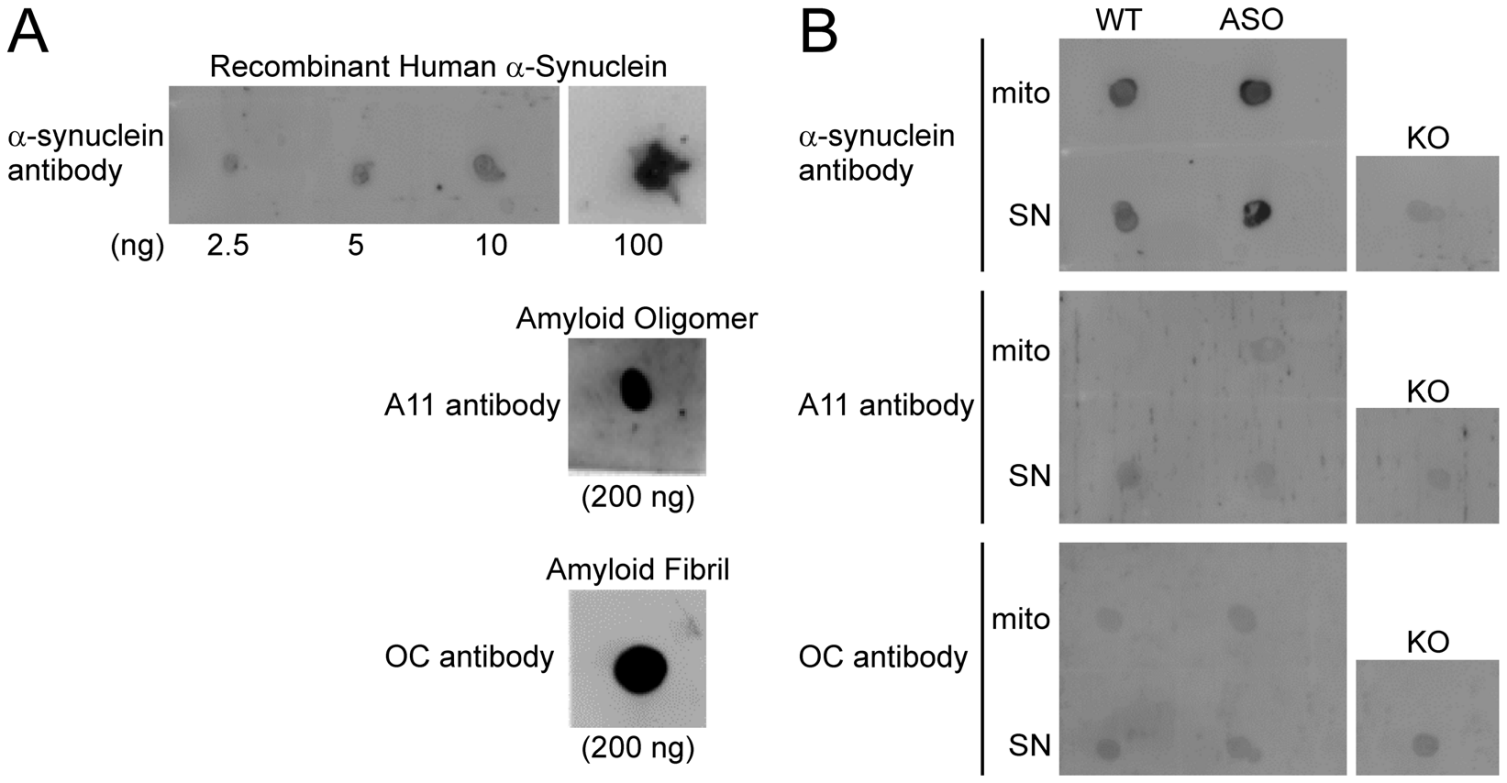
Amyloid-like soluble oligomers or fibrils of elevated human α-synuclein were *not* detected in ASOTg brain mitochondria and synaptoneurosome fractions. A. On dots blots, the α-synuclein antibody detected recombinant human α-synuclein in increasing amounts (2.5–100 ng), serving as a monomer positive control (top panel). Aβ_42_ soluble oligomers and Aβ_42_ fibrils (200 ng each) served as positive controls for the A11 antibody (middle panel) and the OC antibody (bottom panel) respectively. B. The α-synuclein antibody detected elevated human α-synuclein in ASOTg mitochondrial (mito) and synaptoneurosome (SN) fractions (1 µg) relative to WT fractions (top panel). An SN fraction from *Snca^−/−^*mice (KO) served as a negative control. The A11 and OC antibodies failed to detect endogenous mouse (WT) or elevated human α-synuclein (ASOTg) in all fractions when compared to KO negative control (middle/bottom panels).

It cannot be completely ruled out that the 50–60 kDa band together with larger forms observed on Native-PAGE alone may correspond to soluble amyloid-like oligomeric and more aggregated forms of human α-synuclein. To address this possibility further, we used amyloid conformation-selective antibodies (A11, OC)[46] to ask if overexpressed human α-synuclein forms amyloid-like, β-sheet-rich oligomers in brain mitochondria fractions. Because the amyloid-selective antibodies did not detect specific immunoreactive bands resolved by Native-PAGE/Western blots (unpublished observations), we used dot blots exclusively for these experiments (repeated twice). As a positive control for soluble α-synuclein monomers, the COOH terminus #1 antibody detected recombinant human α-synuclein at increasing amounts (Fig. 5 A, top panel). As a positive control for amyloid conformations, the A11 antibody (middle panel) and the OC antibody (bottom panel) detected amyloid-like oligomers and fibrils of Aβ_42_ peptides respectively. The α-synuclein antibody detected over-expressed human α-synuclein in both ASOTg brain mitochondrial and forebrain SN fractions (Fig. 5B, top panel). A SN fraction from *Snca^−/−^*α-synuclein mice (KO) served as negative control for background staining. However, the amyloid conformational antibodies, both the A11 antibody (oligomers, middle panel) and the OC antibody (fibrils, bottom panel), did not detect overexpressed human α-synuclein in ASOTg fractions or the endogenous mouse protein in WT fractions. These results together with the previous Native-PAGE experiments (Fig. 4) were most consistent with soluble monomeric α-synuclein as the major protein form interacting with brain mitochondria.

### Posttranslational Modifications in Brain Mitochondrial Human α-Synuclein

**Figure 6:**
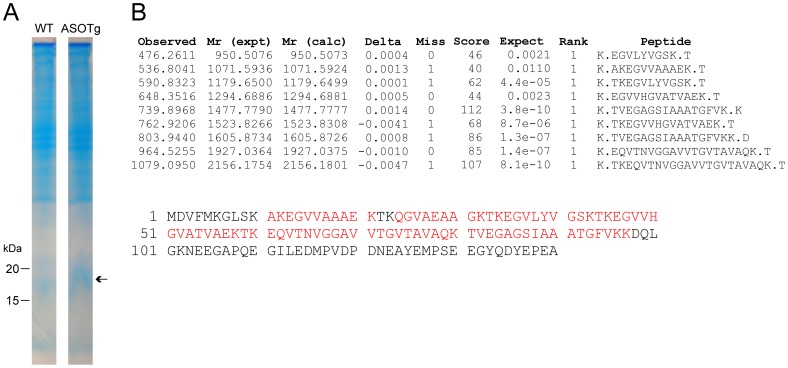
Unmodified human α-synuclein was the major isoform detected in an ASOTg cortex mitochondrial fraction by bottom-up mass spectrometry. A. Distinct blue band at 15–20 kDa (arrow) was detected in the ASOTg lane relative to WT by SDS-PAGE/Coomassie blue staining of cortex mitochondria (250 µg). B. An in-gel, trypsin-digested band was interrogated by nano-chromatography with tandem MS (7 tesla LTQ-FT) to produce partial amino acid sequence for multiple mitochondrial proteins within this size range. The major hits were peptides covering ∼ 60% of the human α-synuclein 140 amino acid sequence (sp|P37840|, NCBI)(shown in red). Precursor masses were screened at high-resolution showing agreement between measured and calculated assignments at the 3^rd^ decimal. Tandem MS data was used to interrogate the presence of human α-synuclein in multiple protein data bases using Mascot software (Matrix Sciences). Searches included the following postranslational modifications (PTMs): Nitro (Y), Oxidation (M), Phosphorylation (ST), Phospho (Y), Sumo (K), ubiquitin: GlyGly (K,S,T).

To search for potential human α-synuclein PTMs in ASOTg mitochondria, we used a combination of bottom-up and top-down MS approaches. Overall the bottom-up approach is used for the detection and identification of expressed proteins along with low stoichiometry PTMs, while the top-down approach is most effective in defining the complete structure of an expressed protein with high stoichiometry PTMs [47]. Using a bottom-up approach, an ASOTg mitochondrial fraction was first resolved by SDS-PAGE (Fig. 6A). The broad band corresponding to the approximate location of the α-synuclein monomeric protein (∼ 15–20 kDa) was excised from the gel, trypsin digested, and subjected to nano-chromatography and tandem MS (nLC-MSMS). The tandem MS data was used to interrogate the presence of human α-synuclein tryptic peptides in a mouse protein data base supplemented with the human α-synuclein sequence, using Mascot software.

**Figure 7:**
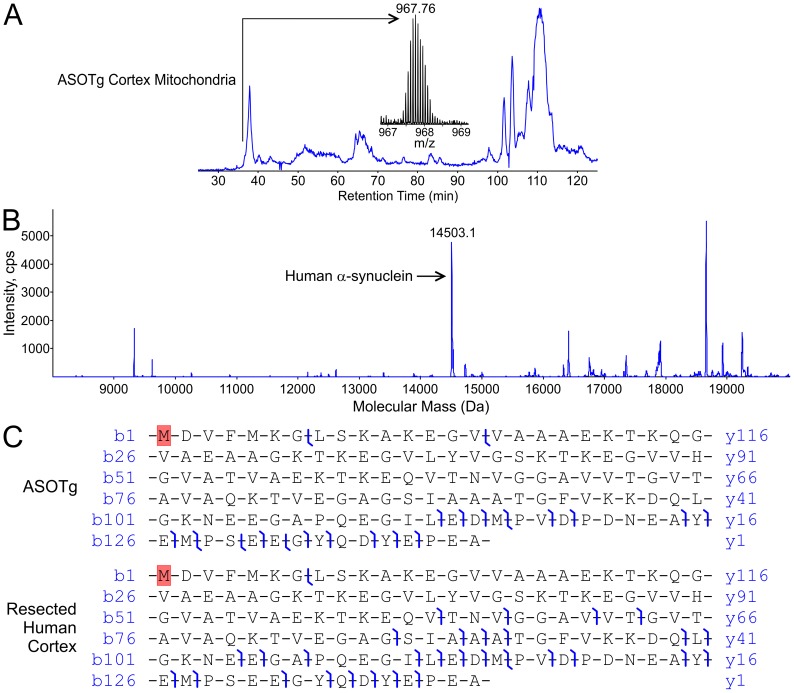
N-terminally acetylated full-length human α-synuclein was the major isoform detected in a ASOTg cortex mitochondrial fraction and in human brain by top-down high-resolution mass spectrometry. A. Total ion chromatogram is shown for the primary separation and the ion-isolation mass spectrum (m/z 967.76) of a candidate full-length α-synuclein of mass 14503.1 Da found in the 37-minute fraction for a ASOTg cortex mitochondrial fraction (500 µg). B. Static nano-electrospray and MSMS displays peaks of various intensities for the average mass spectrum in the same 37-minute fraction (arrow, unmodified human α-synuclein sequenced in panel C). No masses corresponded to known PTMs for human α-synuclein, e,g, nitration, phosphorylation or ubiquitination. C. Hybrid linear ion-trap FT-MS (LTQ-FT) generates high resolution map of product ions formed upon collisionally activated dissociation (CAD) of the m/z 967 precursor, matched at 10 ppm tolerance for confident assignment of the primary amino acid sequence of full-length human α-synuclein from ASOTg mouse (upper panel). Monoisotopic mass of human α-synuclein from ASOTg mouse was 14493.2591 Da (mean of 4 measurements on 4 different ions). A protein of similar mass was recovered from resected human brain and CAD of the m/z 1209 precursor gave a broadly similar product-ion map (lower panel). Monoisotopic mass of human α-synuclein from human brain was 14493.2592 Da (mean of 2 measurements on 2 different ions). The probability that either species analyzed was incorrectly assigned was calculated to be 9.9×10^−31^ (ASOTg) and 4.4×10^−43^ (human brain) using the ProsightPC algorithm at a tolerance of 10 ppm. Note that the top-down approach yields several product ions in the C-terminal region that was poorly covered in the bottom-up experiment (Fig. 6). The species analyzed is shown with N-terminal acetylation of starting methionine (in red) yielding agreement of measured and calculated masses within 10 ppm.

As shown in Figure 6B, the major peptides detected in the total digest covered ∼ 60% of the 140 amino acid sequence of human α-synuclein. The first 10 NH_2_ terminal amino acids and the last 43 COOH terminal amino acids were not represented due to the tryptic digestion pattern (but see Fig. 7) that resulted in peptides either too small or too large for recovery and analysis by nLC-MSMS. Despite appropriate data mining, no PTMs were detected in each human α-synuclein peptide including previously reported sites of tyrosine nitration, methionine oxidation, serine/threonine phosphorylation, tyrosine phosphorylation, lysine SUMOylation, and lysine ubiquitination.

While bottom-up MS analysis of peptides provides a highly sensitive means to detect all covalent modifications that alter mass, the top-down MS approach provides a more balanced overview of the general modification status of the intact protein without the need for tryptic digestion, and the opportunity to detect previously unknown PTMs. For example, major amino acid sites of phosphorylation and truncation reside in the last 43 amino acids of human α-synuclein, which were not represented in tryptic peptides detected by bottom-up MS (Fig. 6). To query the presence of both NH_2_ and COOH terminal tryptic peptides lost in the bottom-up approach, we used the top-down MS approach to determine the full-length 140 amino acid sequence of the intact human α-synuclein protein (Fig. 7). Firstly an intact protein mass spectrum of a candidate full-length human α-synuclein was located in an ASOTg cortex mitochondrial total ion chromatogram (TIC) from the LC-MS+ analysis (Fig. 7A). The de-convoluted mass spectrum is shown (Fig. 7B). The 14503.1 Da species was the best candidate for human α-synuclein, whose calculated average mass including N-acetylation and no other modifications is 14502.17 Da. None of the other peaks’ masses shown, or in the rest of the LC-MS+ experiment, corresponded to those of human α-synuclein containing known PTMs.

The LC-MS+ fraction corresponding to the major peak was subjected to high-resolution top-down MS to generate a map of product ions that allowed a highly confident assignment of the primary amino acid sequence of full-length human α-synuclein including N-terminal acetylation (Fig. 7C, ASOTg, top panel). A near identical high-resolution ion map and full-length amino acid sequence was obtained with a second ASOTg cortex mitochondrial fraction (not shown). Importantly the top-down approach gave several ions resulting from bond cleavages in the COOH-terminal region that were poorly covered (last 43 amino acids) in the bottom up experiment. No PTMs were evident save for the starting methionine residue’s acetylation as reported previously [48]. Interestingly recent reports show that N-terminal acetylation is critical for forming α-helical domains in a purified recombinant form of α-synuclein and can favor oligomer formation [49], [50]. Trypsin digestion of TIC fractions (#35–43, Fig. 7A) followed by bottom-up sequencing using tandem MS yielded similar results to the in-gel digestion experiment (shown in Figure 6), and failed to detect additional PTMs in mitochondrial human α-synuclein in the peak or adjacent fractions (not shown). Other PTMs may be present but below our limit of detection.

As a control for bona-fide human α-synuclein, a similar LC-MS+ fraction was also obtained from surgically resected human cortex from a young non-PD patient and subjected to top-down MS high-resolution analysis (Fig. 7C, bottom panel) as described above. Precursor ion mass was in agreement with the ASOTg sample, and product-ion coverage was again generally confined to the C-terminal region, though somewhat more comprehensive ion coverage was observed in this experiment due to a higher concentration of protein in the sample analyzed. These data support faithful expression of full-length human α-synuclein in the ASOTg mouse.

**Figure 8:**
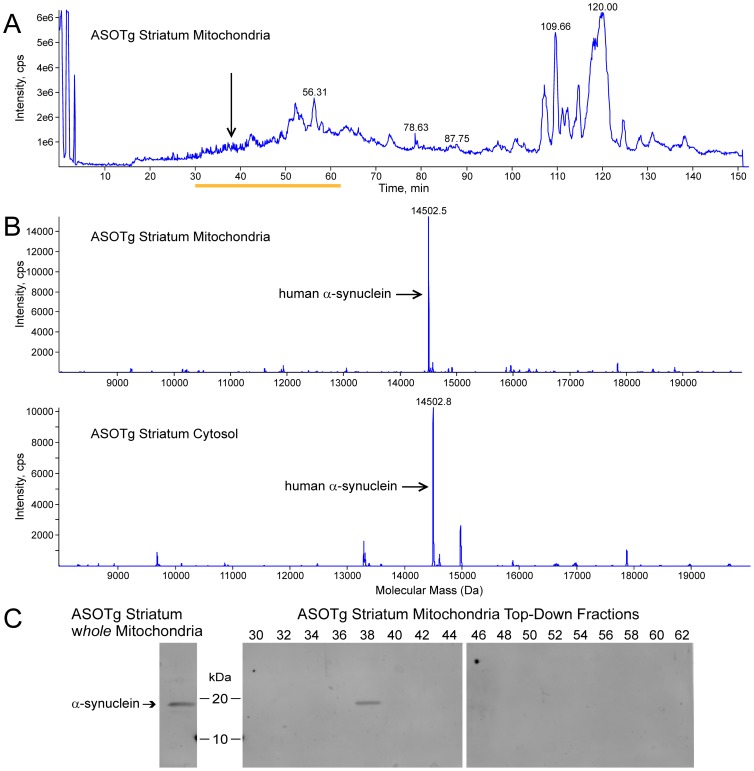
Unmodified full-length human α-synuclein was the major isoform detected in an ASOTg striatum mitochondrial and cytosolic fractions by combined LC-MS+ and Western immunoblotting. A. The total ion chromatogram of LC-MS+ for an ASOTg striatum mitochondrial fraction (250 µg) is shown. Arrow indicates likely human α-synuclein fraction (∼37–38) based on previous top-down MS with ASOTg cortex mitochondria (see Fig. 7). B. Static nano-electrospray and MSMS resolved single fraction (arrow in panel A, 37.55 minutes retention time from total ion chromatogram, comparable to #38 in panel C) from ASOTg striatum mitochondria (arrow in top panel, 14502.5 Da) and a matching cytosolic fraction (arrow in bottom panel, 14502.8 Da) from the same purification procedure. C. As a positive control, human α-synuclein is detected as a major band of 15–20 kDa and a minor smaller band in whole unfractionated ASOTg striatum mitochondria (5 µg) by SDS-PAGE and Western immunoblotting using an antibody selective for human α-synuclein (left panel). Individual top-down intact fractions (#30–62, yellow underline in panel A) were further examined by Western immunoblotting (right panel). Immunoreactivity for unmodified human α-synuclein is detected in fraction #38, but no additional bands corresponding to covalently modified forms that would change relative migration (e.g. truncation, phosphorylation, ubiquitination) are detected.

Based on the previous cortex experiments, ASOTg striatum mitochondria were also resolved by LC-MS+ and a likely fraction (vertical arrow) containing human α-synuclein was isolated (Fig. 8A). The mass spectrum of this mitochondrial fraction and a matching cytosolic fraction are displayed in Fig. 8B (top panel, mitochondria, 14502.5 Da; bottom panel, cytosol, 14502.8 Da). The major peak’s masses (arrows) were consistent with the N-acetylated full-length α-synuclein, while none of the other peaks’ masses corresponded to the mass of human α-synuclein containing PTMs. Individual ASOTg striatum mitochondrial retention fractions (#30–62 minutes, yellow underline)(Fig. 8A) were further examined by Western immunoblotting (Fig. 8C). Only a single band of ∼15 kDa is detected in the 38 minute fraction, corresponding to the approximate fraction containing mostly unmodified human α-synuclein (37.55 minute fraction) (see vertical arrow, Fig. 8A; arrows, Fig. 8B). Although there were peaks of 9–12 kDa in size of potential truncated forms, no smaller bands were detected in fraction #38 nor were larger bands (for example, ubiquitinated) detected in any other fraction on the immunoblot. Thus the combined MS data detect few covalent modifications (except for N-acetylation) of over-expressed human α-synuclein in either mouse brain mitochondrial/cytosolic fractions or in resected human cortex.

## Discussion

### Structural Nature of Overexpressed Mitochondrial Human α-Synuclein

We examined the structural and functional interactions of human α-synuclein with brain mitochondria obtained from adult mice over-expressing the protein [31]. Human α-synuclein was enriched in brain mitochondrial fractions (cortex, striatum) from ASOTg mice and a human cortex homogenate, appeared to have mainly monomeric properties, and had few detectable PTMs save for N-terminal acetylation and some minor carboxyl truncation in mitochondrial fractions. A similar sized protein was also found along with truncated products in synapse-enriched SNs containing elevated human α-synuclein. Moreover a native human α-synuclein protein isoform in the 50–60 kDa range was further cross-validated by its detection in a purified recombinant preparation and in both surgically removed and postmortem human brain tissues.

Interestingly there are reports suggesting that a soluble form of α-synuclein similar in size (∼ 60 kDa) may exist as an α-helical tetramer and may need to destabilize to form disordered monomers prior to forming more toxic soluble amyloid-like oligomers and pre-fibrils [43], [44], [49]. This novel idea, though, continues to be challenged as evidenced by a subsequent report favoring instead aberrant mobility of monomeric α-synuclein on Native-PAGE [45]. The latter results are more in keeping with our own studies here. Based on 2-dimensional PAGE (Native/SDS) experiments, in all likelihood brain mitochondria are impaired mainly by a soluble monomeric form and, to a lesser extent, carboxyl terminally truncated forms of overexpressed human α-synuclein [51], [52]. Perhaps released terminal peptide fragments (10–20 amino acids in length) of human α-synuclein might also be toxic to brain mitochondria at low doses.

Previous studies have detected proteinase K-resistant aggregates of α-synuclein in multiple brain regions of adult ASOTg mice, suggestive of an amyloid conformation for over-expressed human α-synuclein [34], [53]. Based on immunoblotting studies with a limited number of conformation-selective antibodies, human α-synuclein does not appear to assemble into soluble amyloid-like oligomers or fibrils in either brain mitochondria or in synapse-enriched synaptoneurosomes. Overall it appears that a threshold amount of a non-amyloid, monomeric form of human α-synuclein is sufficient to disrupt brain mitochondrial function in adult mice.

### Disruption of Mitochondrial Function by Overexpressed Human Synuclein

Consistent with previous reports [29], [54], the membrane potential is compromised in ASOTg brain mitochondria, while oxidative stress vis á vis ROS generation was observed in these same brain regions. Surprisingly over-expressed human α-synuclein does not selectively interact with any of the mitochondrial inner membrane complexes cI–cV. These results contrast with previous reports showing α-synuclein’s selective interaction with c1 in PD postmortem brain tissue [18] and in brain of PD mouse transgenic models [23], [24].

One possibility is that a copious pool of cytosolic human α-synuclein can stochastically bind to anionic phospholipids in the mitochondrial membrane via its NH_2_ terminus [8]. In essence, this event can randomly coat the mitochondrial membrane, thereby sterically hindering the membrane permeability pore and other vital mitochondrial transport processes. Based on electron microscopy of ASOTg midbrain sections [30], some of this pool is also likely transported into forebrain mitochondria via preferred interactions with cardiolipin-enriched inner membrane segments [8], [55]. Thus it is likely that c1–cV complexes are exposed to human α-synuclein and may contribute collectively to ROS generation. Neuroprotective effects of α-synuclein on mitochondrial functions may also be at play and may help to dampen ROS generation [56], [57].

### Few PTMs in Mitochondrial Human α-Synuclein

Sporadic forms of PD have been closely associated with PTMs of the mature α-synuclein protein [15]. Surprisingly few detectable PTMs were found in mitochondria-associated human α-synuclein or in endogenous α-synuclein contained in surgically resected human cortex using a combination of immunoblotting and MS proteomic approaches. As described above, carboxyl terminally truncated forms of overexpressed human α-synuclein were detected on immunoblots in both ASOTg brain SNs and mitochondria consistent with similar observations in whole brain studies [37]–[39]. However, a recent report suggests that these truncated forms could also correspond to alternatively spliced forms of human α-synuclein in the ASOTg mice [58]. It is curious that both resected human cortex and PD-related postmortem midbrain homogenates (this study) as well as multiple human cortex SN fractions in our previous study [59] did *not* show detectable truncation. Possibly truncation of α-synuclein is concentration dependent and is not selective to PD as suggested by a recent report [60].

In addition to carboxyl terminal truncation, a top-down MS proteomic approach detected N-terminal acetylation in both human α-synuclein overexpressed in mouse brain (ASOTg) mitochondria and endogenous α-synuclein in resected human cortex. Since N-terminal acetylation may favor α-helical N-terminal formations [49], [50], the 50–60 kDa size native protein species that was associated with both mitochondria and synaptoneurosome fractions as well as human brain tissue could also possess some α-helix properties.

PTMs as phosphorylations were not detected in either ASOTg mitochondria or human brain tissue, though a previous report did detect serine^129^ phosphorylation in the ASOTg brain homogenates using a phospho-specific antibody and a cocktail of protein phosphatase inhibitors [61]. Per protocol instructions [62], we did not include phosphatase inhibitors in our mitochondria isolations. Thus the lack of detectable serine^129^and other serine/threonine/tyrosine phosphorylations could be explained to some extent by omission of protein phosphatase inhibitors. However, it is important to point out that a similar top-down MS analysis of a human brain sample from a young non-PD patient, containing both protease and phosphatase inhibitors, yielded a nearly identical high resolution ion map for full-length human α-synuclein, again with no PTMs save for N-terminal acetylation. To our knowledge, this is the first report of the primary amino acid structure of human α-synuclein endogenous to human brain surgically removed from a patient. Thus PTMs appear to occur infrequently in young human brain but may increase progressively with age in PD brain [15]. This observation may also explain why so few PTMs were found in brain mitochondria from the ASOTg mouse, since it is primarily an early model for PD [11], [12], [32]–[34]. Nonetheless N-terminal acetylation and minor carboxyl truncation were the principal PTMs detected in human α-synuclein when associated with brain mitochondria in adult mice.

*In summary*, we have shown that an overexpressed full-length, N-terminally acetylated mostly monomeric form of human α-synuclein was sufficient to disrupt adult brain mitochondrial function. In future experiments, the top-down MS approach described here offers a unique template to obtain the full-length primary amino acid structure and potential PTMs of human α-synuclein present in postmortem PD tissue and, when possible, in surgically resected PD-related tissue at different ages. Overall brain mitochondrial changes appear fairly minimal in the ASOTg mouse, which is already symptomatic at adult ages (2–4 months) but exhibits few additional phenotypic changes save for some dopamine loss at older ages [35]. In light of recent evidence that α-synuclein travels between neurons and possibly between brain regions in PD [63], future experiments should focus on potential dynamic changes in the structures (PTMs, monomeric, oligomeric, fibril) and functions of mitochondrial human α-synuclein in brain regions over the full time course of PD.

## Materials and Methods

### Animals

WT and ASOTg mice over-expressing human α-synuclein under the control of the mouse Thy-1 promoter were generated previously on the C57BL/6 X DBA2 genetic background [31]. Some mice were backcrossed into the C57BL/6 background. Non-Tg α-synuclein control mice (*Snca^+/+^*) and “knock-out” (*Snca^−/−^*) (KO) littermates lacking α-synuclein gene expression were generated previously in the 129 X SvEv genetic background [9]. For all experiments, mice were male and ranged in age from 2–4 months. Groups of 3–4 animals were maintained in cages on a 12 h light cycle at room temperature (21°C) and were fed food and water *ad libitum*. All efforts were made to minimize the number of animals used and their suffering. Studies were carried out according to guidelines of the National Institutes of Health Guide for Care and Use of Laboratory Animals (NIH Publications No. 80–23), “Guidelines for the Use of Animals in Neuroscience Research” (Society for Neuroscience), and with approval from the Institutional Animal Care and Use Committee at UCLA.

### Antibodies/Synthetic Proteins/Mitochondrial Reagents

Primary antibodies were selective for: human α-synuclein NH_2_ terminus (raised against amino acid residues 1–30, clone EP1646Y, rabbit monoclonal, 1∶5,000, EMD/Millipore, Darmstadt, Germany; Temecula, CA, USA), human α-synuclein COOH terminus #1 (raised against amino acid residues 111-131, rabbit polyclonal, 1∶2,500-1∶15,000, EMD/Millipore), human α-synuclein COOH terminus #2 (raised against amino acid residues 111–140, rabbit polyclonal, 1∶500, ABGENT, San Diego, CA, USA); nuclear pore complex proteins (rabbit polyclonal, 1∶500, abcam, USA); ATP synthase (complex V) subunit α (mouse monoclonal, 1∶1,000–1∶10,000, MitoSciences Inc, Eugene, OR), MitoProfile®total OXPHOS antibody cocktail (1∶500, MitoSciences Inc), A11 antibody for amyloid oligomers (rabbit polyclonal, 1∶500) and OC antibody for amyloid fibrils (rabbit polyclonal, 1∶2,500)(gifts from Asa Hatami, Charles Glabe) [46], [64]. Secondary antibodies were horseradish peroxidase-conjugated (anti-rabbit for polyclonals, anti-mouse for monoclonals) (1∶5,000-1∶10,000, Calbiochem, San Diego, CA).

Synthetic proteins included purified recombinant human α-synuclein (EMD/Millipore) and purified Aβ_42_ oligomers and Aβ_42_fibrils (gifts from Asa Hatami, Charles Glabe). Reagents for mitochondrial function included JC-1, MitoSox Red, MitoTracker Green (Molecular Probes/Invitrogen, Eugene, OR) and carbonyl cyanide p-trifluoro methoxy phenyl hydrazine (FCCP) (Sigma-Aldrich, St. Louis, MO).

### Human Brain Tissue

Resected cortex (central operculum) was obtained from a surgical patient (5.8 years old, female with mild neuronal disorganization, ILAE Cortical Dysplasia I) [65], who underwent neurosurgery at the UCLA’s Pediatric Epilepsy Surgery Program [59]. The research protocols were approved by the Institutional Review Board of the Human Protection Research Committee at the University of California, Los Angeles (UCLA). Informed consent to use resected tissue for research purposes and Health Insurance Portability and Accountability Act (HIPAA) authorizations were obtained from parents and legal guardians.

Frozen postmortem human midbrain tissue was obtained from The Neuropathology Core/Brain Bank of the NIA funded UCLA Alzheimer Disease Research Center (Mary Easton Alzheimer Disease Center). Patient demographics were organized according to gender (M, F), age (yrs), diagnosis (control, neuropathology e.g. PD), postmortem interval (hrs) [66]. The following cases were studied: Case #1 (F, 63 yrs, control, 28 hrs), Case #2 (M, 85 yrs, control, 36 hrs), Case #3 [F, 77 yrs, Diffuse Lewy Body Disease (DLBD) + PD, 15 hrs], Case #4 (M, 80 yrs, PD, 3 hrs), Case #5 [F, 85 yrs, Alzheimer’s Disease (AD) IV–V + DLBD, 3 hrs].

### Synaptoneurosome and Mitochondria Preparations

Synaptoneurosome Fractionation: Synaptoneurosome (SN) fractions were isolated from freshly dissected mice half-forebrains (50–200 mg wet weight) in a modified Krebs-Henseleit (mKRBS) buffer pH 7.4 [118.5 mM NaCl, 10 mM Dextrose, 4.7 mM KCl, 1.18 mM KH_2_PO_4_, 1.18 mM MgSO_4_, 24.9 mM NaHCO_3_, 2.5 mM CaCl_2,_ adenosine deaminase (10 µg/ml), benzamide (5 mM), protease inhibitors (leupeptin,1 mg/ml; pepstatin A, 0.005 mg/ml; aprotinin, 0.1 mg/ml), protein phosphatase inhibitors (Na_4_P_2_O_7_, 2 mM; Na_2_MoO_4_, 0.08 mM)] as described previously [11], [36], [59], [67]. SNs were stored at –80°C prior to use in experiments.

Mitochondria Isolation: Brain mitochondrial fractions were isolated from mouse brain regions (cortex, striatum, or cerebellum) by the MITOISO1 protocol (Sigma-Aldrich, St. Louis, MO) [62]. Brain tissue (cortex, striatum cerebellum) (100–150 mg) was freshly dissected into 1.5 ml or 2.0 ml microfuge tubes on ice, washed thoroughly with cold isotonic buffer (10 mM HEPES pH 7.5, 200 mM mannitol, 70 mM sucrose, 1 mM EGTA), and homogenized vigorously with ∼ 10–20 volumes of isotonic buffer plus albumin (2 mg/ml) using a hand-held pestle grinder (Fisher Scientific, Pittsburg, PA). Homogenates were centrifuged at low speed (∼600 x g, 5 min., 4°C) to pellet nuclei and cellular material. The supernatant was centrifuged at high speed (11,000 g, 10 min) to pellet mitochondria. Differential centrifugation was repeated to generate a final mitochondrial enriched pellet that was re-suspended in 20–100 µl storage buffer (10 mM HEPES, pH 7.4, 250 mM sucrose, 1 mM ATP, 0.08 mM ADP, 5 mM sodium succinate, 2 mM K_2_HPO_4_, 1 mM DTT).

For additional purification, cortex mitochondria (300 µg) were prepared fresh by the MITOISO1 protocol and isolated by density gradient centrifugation. Crude mitochondria were loaded on the bottom of an empty ultracentrifuge tube and a step gradient of 1.204 g/ml (bottom), 1.175 g/ml (middle), 1.078 g/ml (top) OptiPrep(iodixanol) (Sigma-Aldrich, St. Louis, MO) in MITOISO1 storage buffer was gently layered above the mitochondria. Tubes were centrifuged at 50,000 x g in a TLS-55 swinging bucket rotor (4 hrs, 4°C) as described [68]. Mitochondria were ‘floated” to an upper major band near the interface of the top/middle densities, carefully removed, pelleted by centrifugation at 11,000 x g, and resuspended in storage buffer (20–50 µl).

### Immunoblotting

Western blotting: For SDS-PAGE experiments, SNs (5–20 µg), and mitochondria/cytosolic fractions (5–25 µg) were homogenized in loading buffer [0.25 M Tris-HCl pH 6.8, 2.2% (w/v) SDS, 10% (v/v) glycerol, 1% (v/v) β-mercaptoethanol, bromophenol blue], boiled, resolved on gels (5% stacking gel, 12% resolving gel) with either large Protean II or mini-Protean Tetrad gel apparatus (Bio-Rad, Hercules, CA) and immunoblotted on polyvinylidene fluoride (PVDF) membranes as previously described [11], [69]. Blots were blocked alone in either 4% milk/phosphate-buffered saline [11] or in 5% milk/Tris-buffered saline with 0.05% Tween-20 (TBST), followed by primary and secondary antibody incubations in the same milk-containing buffer.

For Native-PAGE experiments, mouse forebrain SN fractions (10–25 µg) and mitochondrial/cytosolic fractions from cortex, striatum and cerebellum were resolved together with human brain tissue (surgically resected, postmortem control/PD tissue) (20 µg). Pre-frozen human brain samples were homogenized in mKRBS buffer and filtered once through a nylon mesh as described [59]. Samples were sonicated (3×20 sec) in non-denaturing, non-reducing loading buffer [0.25 M Tris-HCl pH 6.8, 10% (v/v) glycerol, bromophenol blue], centrifuged (1000 x g, 4 min) to remove insoluble material, and loaded without boiling directly onto gels (5% or 10% stacking, 12% resolving). No SDS was present in either gels or electrophoresis buffer prior to immunoblotting as described [70]. Recombinant human α-synuclein (0.2–0.4 µg), sonicated in 10 mM Tris-HCl pH 7.4, served as a monomer control.

PVDF membranes were probed with primary antibodies followed by horseradish peroxidase-conjugated secondary antibodies (anti-rabbit for polyclonals, anti-mouse for monoclonals) (1:10,000). Bound antibodies were visualized by ECL-Plus fluorescence (GE Healthcare Life Sciences, Piscataway, NJ); fluorescent images were acquired using the Typhoon 9410 Imaging System (GE Healthcare) in the UCLA Biological Chemistry Imaging Facility. Quantitative analysis was performed as needed with Imagequant 5.2 software by Molecular Dynamics (Sunnyvale, CA).

Both brain mitochondrial and recombinant human α-synuclein were analyzed by two-dimensional PAGE (Native/SDS). Mitochondrial aliquots (20 µg protein) were mixed with an equal volume of non-denaturing, non-reducing sample buffer (see Native-PAGE above). Samples were maintained at room temperature, sonicated, and centrifuged to remove insoluble material as described previously. Following Native-PAGE in the first dimension (1D), appropriate lanes were dissected from the gel and incubated with SDS sample buffer for 30 min at 37°C. The lanes were inserted horizontally onto the SDS-PAGE gel for the second dimension (2-D) and secured to the stacking gel with a solution of heated 1% agarose in SDS-containing buffer lacking bromophenol blue. A well for protein standards was formed in the agarose solution adjacent to the native gel segment using a plastic insert during agarose cooling. SDS PAGE, Western transfer and immunostaining were performed as described [11], [69].

Dot Blotting: The α-synuclein protein was examined by dot blot using antibodies selective for human α-synuclein protein or amyloid-like oligomers/fibrils. Aliquots (1–2 µl; 1 µg each) of cortex mitochondria or forebrain SN preparations were spotted onto nitrocellulose membranes (0.2 µm), dried at room temperature, and blocked with 5% milk in TBST. Blots were incubated overnight at 4°C with antibody selective for human α-synuclein, an amyloid conformation antibody selective for soluble oligomers (A11), or an amyloid conformation antibody selective for soluble fibrils (OC)[46]. Purified recombinant human α-synuclein in 10 mM Tris-HCL, pH 7.4 served as positive control for soluble monomers (2.5–100 ng per spot). The Aβ_42_ oligomers and fibrils (200 ng/spot) on pre-spotted strips served as positive controls for conformations of amyloid oligomers and fibrils.

### Mitochondrial Assays

Mitochondrial Membrane Potential: Mitochondrial preparations were diluted to 1 mg/ml protein with MITOSIO1 storage buffer and were assayed for membrane potential in 96-well plate by mixing 5 µl of mitochondria with 95 µl of assay buffer containing JC-1 (1 µg/ml) [71], [72]. Red fluorescence (Ex  = 530, Em  = 590) was measured at 2, 10, and 20 min using a Cytofluor 2300 fluorescence plate reader (EMD/Millipore). Background values were obtained from wells containing no mitochondria. In pilot experiments, we showed that membrane potential values for freshly prepared versus pre-frozen mitochondria from the same WT brain regions were not significantly different (fresh, 2057±78.7; frozen-thawed, 1930.0±106.0, N = 4 mice, Student *t*-test, P = 0.372). Moreover inclusion of FCCP (10 µM) in separate wells resulted in >95% reduction in mitochondrial membrane potential (fresh, 3.8 ±1.6%; frozen-thawed, 4.9±2.0%, P = 0.91), thereby confirming authentic mitochondrial membrane potential. Thus to conserve precious ASOTg mice, we used both fresh and pre-frozen mitochondria from multiple mice for membrane potential and ROS assays.

Mitochondrial Reactive Oxygen Species (ROS): ROS produced by mitochondria was measured using MitoSox Red (Molecular Probes/Invitrogen) as described [72]. Mitochondrial preparations were diluted to 1 mg/ml protein with storage buffer and 5 µl was added to 95 µl assay buffer containing 4 µM MitoSox Red and 0.2 µM MitoTracker Green (Molecular Probes/Invitrogen). Measurements of red (530/590) and green (485/530) fluorescence were taken at 2, 30, and 60 min using a fluorescence plate reader. Red fluorescence measurements were normalized to MitoTracker Green fluorescence representing total mitochondrial mass.

Mitochondrial Electron Transport Complex I–V Immunocapture: Mitochondria (input), isolated by the MITOSIO1 protocol, were diluted to 5.5 µg/ml protein and incubated with antibody-conjugated beads selective for electron transport complexes I–V and pyruvate dehydrogenase (Mito Sciences Inc). Beads were washed thoroughly with 1 mM lauryl maltoside detergent-containing buffer and bound-protein complexes were eluted with 0.2 M glycine/1 mM lauryl maltoside buffer pH 2.5 and analyzed by SDS PAGE/Western blotting. Blots were initially probed with antibodies to human α-synuclein, washed several times with TBST buffer, and reprobed with a total OXPHOS antibody cocktail selective for each of the 5 electron transport complexes (cI–cV) to confirm selective immunocapture.

### Liquid Chromatography and Mass Spectrometry

Bottom-up MS: Mitochondrial proteins (250 µg) (MITOISO1 protocol) were resolved by SDS-PAGE as described [11]. The gel was fixed in isopropanol solution (25% isopropanol, 15% acetic acid) for 30 minutes at room temperature, stained overnight at room temperature with coomassie solution [0.006% (wt/vol) coomassie blue G-250, 10% acetic acid], and destained overnight with 10% acetic acid as described [73]. A broad band corresponding to the approximate location of the α-synuclein monomeric protein (∼ 15–20 kDa) was excised from the gel, trypsin digested to generate multiple peptides, and analyzed by nano-liquid chromatography combined with tandem mass spectrometry as described [74], [75]. Tandem MS data was used to interrogate the presence of PTMs in human α-synuclein peptides in multiple data bases using Mascot software (Matrix Sciences). PTM searches included: tyrosine nitration [Nitro (Y)], oxidized methionine [Oxidation (M)], serine/threonine phosphorylation [Phospho (ST)], tyrosine phosphorylation [Phospho (Y)], small-ubiquitin-related modifier (SUMOylation) [SUMO (K)], lysine ubiquitination [Ubiquitin: GlyGly (K,S,T)].

Top-down MS: Mouse cortex mitochondria fractions (500 µg) and striatum matching mitochondria/cytosol fractions (250 µg each) were purified by the MITOSIO1 protocol, precipitated with chloroform/methanol, and dissolved in formic acid. For a more precise comparison to the human condition, a sample (250–500 µg) of surgically resected cortex from a human patient (same sample source, Native-PAGE above) was also examined. Pre-frozen human resected brain tissue was solubilized with a pestle in ice-cold homogenization buffer [50 mM HEPES (pH 7.4), 10 mM MgCl_2_, 1 mM EDTA, 1 mM EGTA, 10 mM benzamide, 100 ng/ml leupeptin, 100 ng/ml aprotinin, 0.01% Triton X-100, 2 mM Na_4_P_2_O_7_, 0.08 mM Na_2_MoO_4_] [11], then precipitated and resolved in formic acid as above. Fractions were separated by reverse-phase chromatography with eluent split between a low-resolution electrospray ionization MS and a fraction collector (LC-MS+) to generate a total ion chromatogram (TIC) [76]–[79]. For ASOTg striatum mitochondria, individual retention fractions (minutes), resolved on TIC of striatal mitochondrial intact proteins, were further examined for PTMs by Western immunoblotting with human α-synuclein antibody.

For ASOTg mouse cortical mitochondrial fractions (two separate experiments) and a resected human brain sample, LC-MS+ data directed high-resolution top-down experiments allowing optimal data collection strategies by static nano-electrospray and MSMS. A hybrid linear ion-trap ion cyclotron resonance FT-MS (7 Tesla LTQ-FT Ultra; Thermo) was used to generate a high-resolution measurement of precursor-ion mass and a map of product ions formed upon collisionally activated dissociation (CAD) of candidate precursor ions corresponding to the full-length α-synuclein of mass 14503.1 Da found in the 37-minute LC-MS+ fraction. Monoisotopic precursor-ion masses were calculated using the Xtract algorithm (version 3.9; Thermo) and monoisotopic product-ion masses were calculated and matched to the human α-synuclein primary structure using the ProsightPC algorithm (version 2.0; Thermo). A tolerance of 10 ppm was used throughout.

### Statistical Analysis

Student’s *t*-tests were used for two group comparisons. Appropriately designed Two Way ANOVAs followed by Bonferroni *t*-tests were used for multiple comparisons [11].
